# Acute Pericardial Effusion After Vein of Marshall Ethanol Infusion for Persistent Atrial Fibrillation

**DOI:** 10.1002/joa3.70351

**Published:** 2026-05-05

**Authors:** Hualong Li, Jianlong Yin, Jian Liang, Ning Huang, Teng Li

**Affiliations:** ^1^ Cardiac Arrhythmia Ward Fuwai Hospital Chinese Academy of Medical Sciences Shenzhen China

**Keywords:** low‐voltage area, pericardial effusion, persistent atrial fibrillation, vein of Marshall ethanol infusion

## Abstract

**Background:**

Vein of Marshall ethanol infusion (VOM‐EI) is associated with a higher incidence of pericardial effusion (PE). However, few studies have provided detailed explorations of acute PE following VOM‐EI. This study aims to identify the risk factors for acute PE following VOM‐EI in patients with persistent atrial fibrillation (PeAF).

**Methods and Results:**

We conducted a retrospective analysis of 87 patients who underwent VOM‐EI and catheter ablation. All patients underwent PE screening with ultrasound within 12 h post‐procedure. Left atrial high‐density bipolar voltage mapping was performed before and after VOM‐EI to identify the VOM‐EI related low‐voltage area (LVA). The Boruta algorithm and logistic regression were employed to identify risk factors for PE. The model's performance was assessed using the area under the receiver operating characteristic curve (AUC). Acute post‐procedural PE was observed in 22 out of 87 patients (25.2%); no cases of cardiac tamponade occurred. The mean effusion depth was 4.59 ± 2.72 mm. The Boruta algorithm ranked the importance of variables as follows: LVA, procedure time, and heart failure. Multiple logistic regression analysis identified LVA as an independent predictor for PE (OR: 1.15; 95% CI: 1.01–1.30; *p* = 0.030) after adjusting for procedure time and heart failure. The predictive model for LVA exhibited an AUC of 0.681 (95% CI: 0.538–0.824).

**Conclusions:**

Acute PE is a common finding after combined VOM‐EI and catheter ablation for PeAF. The VOM‐EI‐related LVA was identified as an independent risk factor for PE.

## Graphical abstract

This study reports a 25.2% incidence of acute pericardial effusion following vein of Marshall ethanol infusion for persistent atrial fibrillation. The extent of the resulting low‐voltage area was identified as an independent predictor of pericardial effusion, reflecting the magnitude of chemical injury.

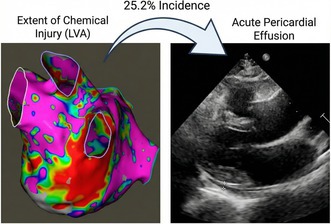

## 1 Introduction

Atrial fibrillation (AF) remains the most common sustained cardiac arrhythmia worldwide, imposing a substantial burden on healthcare systems due to its association with increased risks of stroke, heart failure, and mortality [1]. PeAF presents particular therapeutic challenges due to more advanced electrical and structural remodeling [2]. Pulmonary vein isolation (PVI) is the cornerstone of catheter ablation for AF; however, success rates for PeAF with PVI alone remain modest, often necessitating additional substrate modification strategies [3]. The vein of Marshall (VOM), an embryological remnant of the left superior vena cava, has emerged as an important arrhythmogenic structure in PeAF. It contributes to AF maintenance through its rich autonomic innervation and connection to arrhythmogenic myocardial sleeves. Consequently, VOM ethanol infusion (VOM‐EI) has been developed as an adjunctive technique to radiofrequency ablation, effectively targeting these regions through chemical ablation [4].

However, the unique mechanism of VOM‐EI, which involves the controlled delivery of ethanol into a venous structure adjacent to the pericardial space, creates a distinct risk profile for pericardial complications. A previous study reported that subacute pericardial effusion (PE) or pericarditis occurred in 13 out of 185 patients undergoing VOM‐EI [5]. Moreover, VOM‐EI was reported to provoke PE resulting in a fatal complication in a chronic hemodialysis patient with PeAF [6]. Delayed PE has also been found to be caused by pericarditis after VOM‐EI [7]. Nevertheless, specific data regarding acute PE following VOM‐EI remain limited, particularly concerning the extent of the lesion caused by ethanol infusion.

This retrospective study aims to address these knowledge gaps by comprehensively evaluating the incidence and predictors of acute PE following VOM‐EI in patients with PeAF.

## 2 Methods

During the study period, VOM‐EI combined with radiofrequency ablation was adopted as the standard primary intervention strategy for all consecutive patients presenting with non‐valvular PeAF. From August 2021 to March 2024, 141 consecutive patients were screened. A total of 54 patients were excluded, including 46 due to non‐visualization of the VOM, 3 due to preoperative PE, and 5 due to mechanical complications (specifically, 4 cases of VOM dissection and 1 case of VOM perforation). The remaining 87 patients successfully underwent VOM‐EI and were included in the final analysis. This study was approved by the Ethics Committee of Fuwai Shenzhen Hospital, and all participants provided written informed consent before the procedure.

All procedures were conducted under conscious sedation achieved with fentanyl. Activated clotting time was consistently maintained within a range of 300 to 400 s throughout the procedure. A predefined anatomical ablation strategy was applied for all patients. Procedures were performed using the CARTO3 mapping system (Biosense Webster, CA, USA). High‐density voltage maps of the left atrium (LA) were acquired before and immediately after VOM‐EI under AF using a multipolar mapping catheter (Pentaray, Biosense Webster, CA, USA).

**FIGURE 1:**
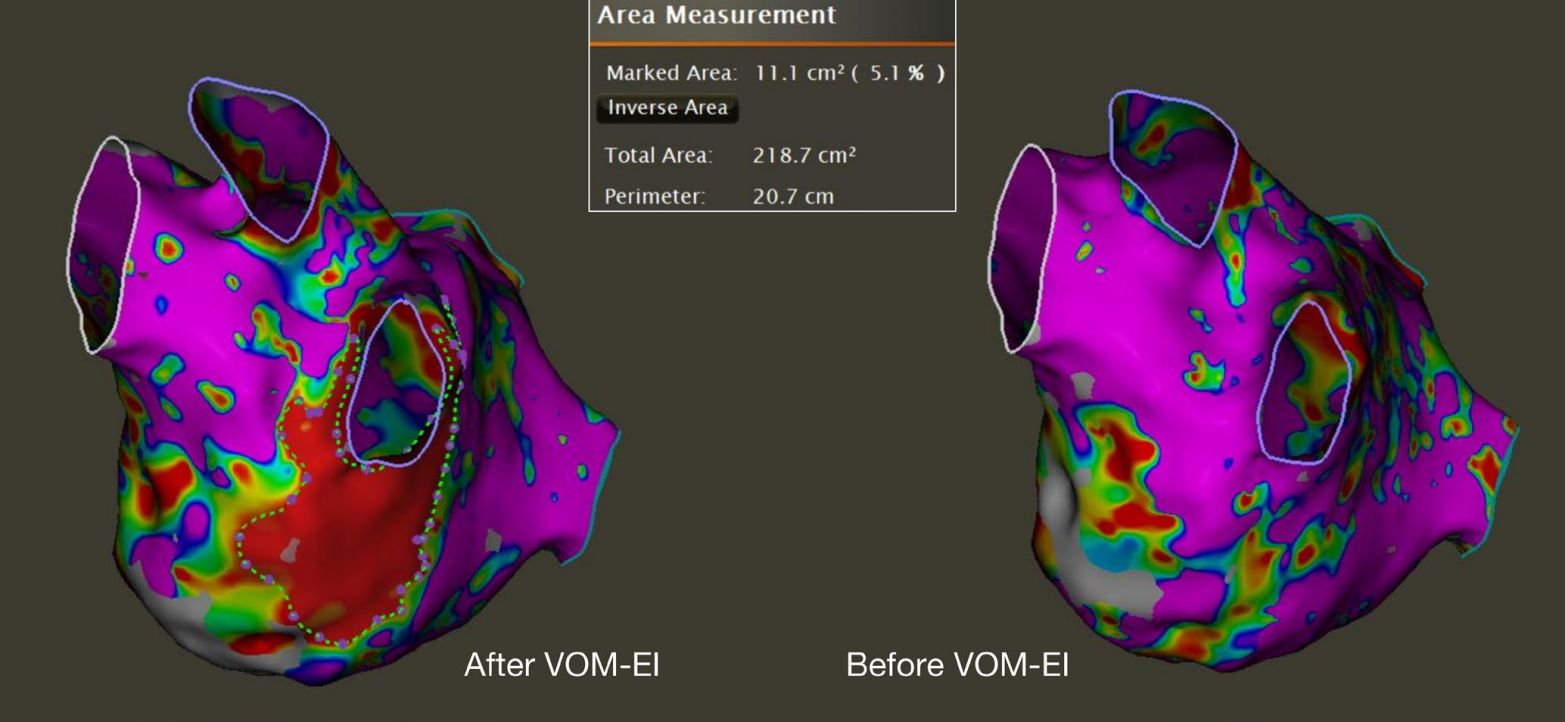
Measurement of LVA resulted from VOM‐EI. A post VOM‐EI high‐density map of the left atrium showed the lesion of LVA. The area measurement tool was used to quantify the lesion's extent. Abbreviations: LVA, low‐voltage area; VOM‐EI, vein of Marshall ethanol infusion.

VOM‐EI procedure was systematically initiated as the primary intervention in the clinical protocol, as documented in the referenced literature [8]. The initial step involved the placement of an 8.5‐French long introducer sheath into the right atrium, with its tip positioned directly caudal to the coronary sinus (CS) ostium. Under fluoroscopic guidance using a right anterior oblique (RAO) projection, a 6F Judkins R4.0 guiding catheter was introduced into the CS. It was then meticulously manipulated to achieve a posterior and superior trajectory adjacent to the Vieussens valve to facilitate the cannulation of the VOM ostium. A selective VOM venogram was acquired at this stage to confirm anatomical positioning. The inability to cannulate the ostium and visualize the vessel via this conventional venogram was the defining criterion for “non‐visualization of the VOM”. Any angiographic evidence of dissection during initial cannulation resulted in procedural abortion. Subsequently, a BMW guidewire (0.014 in. in diameter, 190 cm in length; Abbott Vascular) was advanced into the VOM. This guidewire was supported by an over‐the‐wire balloon catheter (with a diameter range of 1.5–2.5 mm and a length of 8–12 mm; Boston Scientific). The selected balloon was then progressively inflated within the VOM to a maximum pressure of 6–10 atm to achieve complete vessel occlusion. Before VOM‐EI, a 1 mL bolus of contrast medium was administered to verify occlusion and vessel integrity. This step served to rule out mechanical VOM dissection caused by guidewire or balloon manipulation. Patients with verified dissection at this stage were excluded. Following confirmation, 5 mL of absolute ethanol was infused manually with gentle pressure. The infusion rate was controlled at 1 mL/min to avoid acute pressure spikes. Throughout the injection, tactile feedback was monitored: significant increases in resistance (suggesting catheter kinking) or sudden drops in resistance (suggesting balloon instability) prompted immediate suspension of the infusion for re‐evaluation. Post‐infusion selective venography was repeated to assess the initial effect. After a five‐minute waiting period, an additional ethanol injection was administered in an identical manner, targeting the proximal and/or middle segment of the VOM. The volume of ethanol administered was individualized based on the specific VOM anatomy. The total dose was titrated according to the vessel caliber, length, and the extent of arborization visualized on the initial venogram. Larger volumes (up to a maximum of 15 mL) were utilized for patients with a large, dominant VOM or extensive lateral branches to ensure complete impregnation of the target tissue. Immediately following the VOM‐EI procedure, an electroanatomical voltage map of LA was reconstructed. The resulting LVA induced by VOM‐EI were quantitatively analyzed using a dedicated software area measurement tool. These regions were defined as areas exhibiting abnormal voltage (using a cutoff of < 0.1 mV), while deliberately excluding any LVA that had been present prior to VOM‐EI (Figure 1).

Subsequent PVI and linear ablation were executed using an open‐irrigation, contact‐force sensing ablation catheter (ThermoCool SmartTouch SF Catheter, Biosense Webster, CA, USA). Ablation was performed point‐by‐point in power‐control mode, set at 50 W, with a temperature limit of 43°C and an irrigation flow rate of 15 mL/min. Within the coronary sinus, radiofrequency energy was delivered at a reduced power output of 25 W, with an irrigation flow rate of 25 mL/min. The administration of ibutilide was employed to facilitate conversion to sinus rhythm during the ablation process. If pharmacological conversion proved unsuccessful, electrical cardioversion was ultimately performed to restore sinus rhythm. The procedural endpoint was defined as the achievement of both complete PVI and confirmed bidirectional block across all created ablation lines, with confirmation performed during sinus rhythm.

All patients underwent a transthoracic echocardiogram within 12 h following the procedure to screen for the presence of acute PE. Patients were subsequently categorized into two distinct groups based on the presence or absence of a PE. In cases where a PE was identified, medical therapy with diuretics and colchicine was initiated; pericardiocentesis with drainage was performed if deemed clinically necessary.

## 3 Statistical Analyses

Continuous variables with a normal distribution are presented as mean ± standard deviation, while non‐normally distributed data are presented as median (interquartile range). Categorical variables were analyzed using the chi‐square test or Fisher's exact test, as appropriate. Continuous variables were compared using the Welch two‐sample *t*‐test or the Mann–Whitney U test (rank‐sum test). To identify important risk factors for PE, we performed Boruta feature selection using a random forest classifier. This algorithm compares the importance score of each predictor against the importance scores of randomly generated shadow features.

Univariate logistic regression analysis was used to assess the association between each individual factor and PE. Subsequently, multivariate logistic regression analysis was performed to identify factors independently associated with PE, while adjusting for potential confounders. The selected factors were used to construct a prediction model. Model performance was evaluated using the receiver operating characteristic (ROC) curve, with the area under the ROC curve (AUC) ranging from 0.5 (no discriminatory power) to 1.0 (perfect discriminatory power). A *p*‐value of < 0.05 was considered statistically significant. All statistical analyses were conducted using R software (version 4.2.2; R Foundation for Statistical Computing) and MSTATA software (www.mstata.com).

## 4 Results

Of the 87 consecutive patients included in this study (median age 66 years; 72.4% male), 65 (74.7%) did not develop acute PE (PE negative group), while 22 (25.3%) did (PE positive group). There were no cases of pericardial tamponade, and no patient required pericardial puncture. The mean effusion depth was 4.59 ± 2.72 mm. PE depths were 2–5 mm in 77.3% of patients, 6–10 mm in 18.2%, and only one patient had a maximum depth of 12 mm. This patient remained hemodynamically stable and did not require vasopressor support or pericardial intervention.

Baseline characteristics are summarized in Table 1, and procedural data in Table 2. Statistically significant differences were observed between the two groups: patients in the PE positive group had a higher prevalence of heart failure (45.5% vs. 15.4%, *p* = 0.004), higher NT‐proBNP levels (median 862 pg/mL vs. 614 pg/mL, *p* = 0.017), and greater use of loop diuretics (50.0% vs. 12.3%, *p* < 0.001). Additionally, the PE positive group had larger LVA (median 10.2 cm^2^ vs. 7.4 cm^2^, *p* = 0.027). No other significant differences were identified in sex, procedure time, ethanol volume, linear ablation, or CS ablation. Notably, postoperative high‐sensitivity C‐reactive protein levels increased significantly compared to preoperative levels, though no intergroup difference was observed.

**TABLE 1:** Baseline characteristics.

Variables	All *n* = 87	PE negative *n* = 65	PE positive *n* = 22	*p*
Age, years	66 (58, 71)	64 (58, 71)	67 (58, 71)	0.604
Female, *n*	24 (27.6%)	17 (26.2%)	7 (31.8%)	0.607
AT/AFL, *n*	10 (11.5%)	9 (13.8%)	1 (4.5%)	0.441
Hypertension, *n*	44 (50.6%)	32 (49.2%)	12 (54.5%)	0.666
Diabetes, *n*	17 (19.5%)	12 (18.5%)	5 (22.7%)	0.757
Coronary artery disease, *n*	12 (13.8%)	9 (13.8%)	3 (13.6%)	> 0.999
Stroke, *n*	14 (16.1%)	10 (15.4%)	4 (18.2%)	0.745
Heart failure, *n*	20 (23.0%)	10 (15.4%)	10 (45.5%)	0.004
CHA2DS2‐VASc score	2.00 (1.00, 3.00)	2.00 (1.00, 3.00)	2.50 (2.00, 4.00)	0.205
HAS‐BLED score	1.00 (0.00, 1.00)	1.00 (0.00, 1.00)	1.00 (0.00, 1.00)	0.585
Creatinine, mol/L	86 (72, 99)	84 (72, 97)	94 (85, 99)	0.082
NT‐proBNP, pg/mL	672 (476, 1113)	614 (458, 936)	862 (653, 1513)	0.017
Preoperative hs‐CRP, mg/L	1.15 (0.49, 2.25)	1.20 (0.50, 2.15)	1.15 (0.38, 3.22)	0.637
Postoperative hs‐CRP, mg/L	9 (6, 15)	9 (6, 14)	10 (7, 20)	0.571
Echography				
LA diameter, mm	40.8 ± 5.2	40.6 ± 5.0	41.5 ± 5.7	0.476
LVED diameter, mm	46.0 (43.0, 49.0)	46.7 (44.0, 49.0)	45.0 (43.0, 49.0)	0.691
LVEF, %	57 (54, 62)	57 (55, 62)	58 (54, 62)	0.806
Loop diuretics, *n*	19 (21.8%)	8 (12.3%)	11 (50.0%)	< 0.001

Abbreviations: AT, atrial tachycardia; AFL, atrial flutter; hs‐CRP, hypersensitive C‐reactive protein; LA, left atrium; LVED, left ventricular end‐diastolic; LVEF, left ventricular ejection fraction; NT‐proBNP, N‐terminal pro‐B type natriuretic peptide.

**TABLE 2:** Procedural data.

Variables	All *n* = 87	PE negative *n* = 65	PE positive *n* = 22	*p*
LVA, cm^2^	7.6 (5.4, 11.8)	7.4 (4.7, 11.0)	10.2 (7.3, 13.5)	0.027
Ethanol volume, mL	10.0 (8.0, 10.0)	10.0 (8.0, 10.0)	10.0 (10.0, 12.0)	0.074
Procedure time, minute	139 ± 26	140 ± 26	135 ± 26	0.419
MI ablation, *n*	85 (97.7%)	64 (98.5%)	21 (95.5%)	0.444
Roofline ablation, *n*	61 (70.1%)	46 (70.8%)	15 (68.2%)	0.819
CS ablation, *n*	47 (54.0%)	37 (56.9%)	10 (45.5%)	0.351
CTI ablation, *n*	28 (32.2%)	19 (29.2%)	9 (40.9%)	0.311
MI linear block, *n*	77 (88.5%)	57 (87.7%)	20 (90.9%)	> 0.999
Ibutilide, *n*	65 (74.7%)	47 (72.3%)	18 (81.8%)	0.375
Electrical cardioversion, *n*	26 (29.9%)	20 (30.8%)	6 (27.3%)	0.757

Abbreviations: CS, coronary sinus; CTI, cavo‐tricuspid isthmus; LVA, low‐voltage area; MI, mitral isthmus.

**FIGURE 2:**
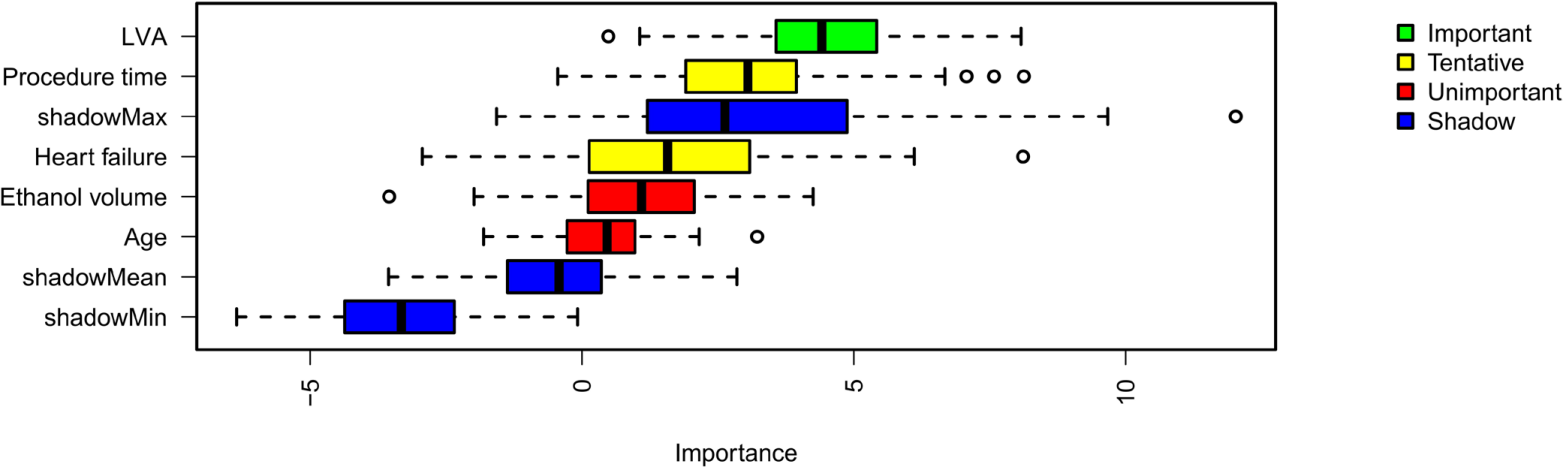
Importance ranking by the Boruta algorithm. The plot demonstrates a boxplot of important attributes in color green, tentative attributes in yellow, non‐important attributes in red, and shadow attributes in blue box, respectively. The vertical axis lists the name of each variable, and the horizontal axis is the Z‐value. Abbreviations: LVA, low‐voltage area.

The original prediction model for acute PE included LVA, age, heart failure, procedure time, and ethanol volume. The Boruta algorithm was applied to the training cohort using a random forest classifier to evaluate variable importance against shadow features. LVA was identified as an “important” predictor, while procedure time and heart failure were “tentative”; age and ethanol volume were deemed “unimportant” (Figure 2).

**FIGURE 3:**
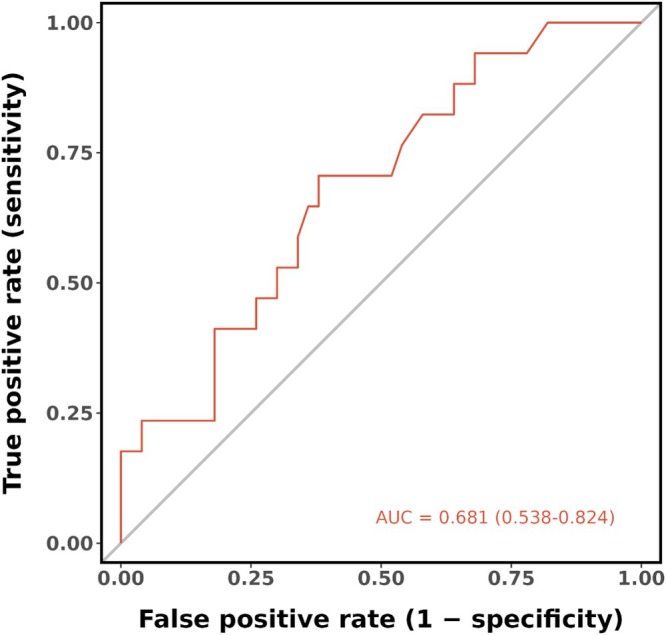
Receiver operating characteristic (ROC) curve analysis. The ROC curve analysis demonstrated that the predictive performance of LVA yielded an area under the curve (AUC) of 0.681, with a 95% confidence interval ranging from 0.538 to 0.824.

Univariate logistic regression indicated that LVA and heart failure were significantly associated with acute PE, whereas age, procedure time, and ethanol volume were not. Multivariate analysis, incorporating LVA, procedure time, and heart failure as selected by the Boruta algorithm, revealed that only larger LVA was independently associated with an increased risk of acute PE (adjusted OR: 1.15; 95% CI: 1.01–1.30; *p* = 0.030), as presented in Table 3. Receiver operating characteristic (ROC) curve analysis showed that LVA had an area under the curve (AUC) of 0.681 (95% CI: 0.538–0.824) for predicting acute PE (Figure 3).

**TABLE 3:** Univariate and multivariate logistic regression analysis.

Variables	Univariable			Multivariable		
	OR	95% CI	*p*	OR	95% CI	*p*
LVA	1.16	1.03, 1.30	0.016	1.15	1.01, 1.30	0.030
Age	1.00	0.96, 1.05	0.840			
Heart failure						
No	—	—		—	—	
Yes	4.58	1.56, 13.45	0.006	2.41	0.60, 9.71	0.216
Procedure time	0.99	0.97, 1.01	0.411	0.99	0.97, 1.01	0.368
Ethanol volume	1.20	0.97, 1.49	0.088			

Abbreviations: CI, Confidence Interval; LVA, low‐voltage area; OR, Odds Ratio.

## 5 Discussion

The principal findings of this study are as follows: (a) acute PE was not uncommon following VOM‐EI and catheter ablation for PeAF, yet it rarely progressed to pericardial tamponade; (b) acute PE was associated with the lesion created by VOM‐EI, wherein a larger VOM‐EI‐related LVA predicted an increased risk of acute PE.

PE and cardiac tamponade remain among the most serious complications of catheter ablation for AF. Traditional radiofrequency ablation carries an estimated risk of 1.2%–2.0% for clinically significant PE. A recent systematic review and meta‐analysis of VOM‐EI procedures reported variable complication rates across studies, with PE being among the most frequently observed adverse events [9]. Ethanol‐induced chemical ablation causes localized necrosis and inflammation, which alters tissue integrity and compromises regenerative capacity. This inflammatory response may extend beyond the endocardial surface to affect the epicardium and pericardium, potentially disrupting microvascular integrity and increasing permeability [10]. Furthermore, the temporal resolution of VOM‐EI‐induced lesions due to progressive edema resolution, as described in recent literature, suggests that the acute inflammatory phase immediately post‐procedure represents a critical window for effusion development [11]. Although a standard 6F guiding catheter was used, we emphasize that gentle catheter manipulation is critical to minimize mechanical stress on the venous endothelium, which could theoretically synergize with ethanol‐induced inflammation.

This study demonstrates a significant incidence of acute PE following VOM‐EI and catheter ablation, with approximately one‐quarter of the cohort developing this complication. Although none of the effusions progressed to tamponade, their frequency underscores the procedural risks associated with this increasingly utilized technique. The postoperative level of high‐sensitivity C‐reactive protein was significantly elevated compared to preoperative levels, indicating an inflammatory response associated with PE. In one study, 22.7% of patients treated with VOM‐EI experienced contrast medium extravasation due to capillary rupture, with two patients requiring additional therapeutic intervention for PE [12]. However, in most patients, pericardial effusions resolved spontaneously by the next day, a finding consistent with our observations. While other studies have focused on subacute clinical PE or pericardial tamponade, our study provides a detailed characterization of acute PE following VOM‐EI.

Post‐procedural voltage analysis represents a straightforward and reproducible method to evaluate the impact of ethanol delivery into the VOM on the newly formed lesion in the LA. The identification of LVA as the most significant variable through Boruta algorithm analysis underscores its central role in the pathogenesis of PE, while the inclusion of procedure time and heart failure status provides a more comprehensive risk assessment framework. Multivariable logistic regression analysis revealed that an increased LVA was significantly associated with a higher risk of postoperative PE, with an odds ratio of 1.15. The predictive performance of our model, demonstrated by an area under the curve of 0.681 in receiver operating characteristic analysis, indicates moderate discriminative ability. The strong association between LVA and PE suggests that the extent of myocardial injury resulting from VOM‐EI may influence the propensity for fluid accumulation in the pericardial space.

Our findings concerning the relationship between ethanol dose and PE risk, though not reaching statistical significance in the multivariable model, warrant further investigation. A potential dose–response relationship is biologically plausible, as larger ethanol volumes may cause more extensive chemical injury and inflammation. A comparative analysis based on ethanol volume was not performed due to the limited variance associated with our standardized 10 mL dosing protocol. Instead, LVA was utilized as the primary indicator of tissue injury, as it integrates the interplay between ethanol volume and vascular anatomy. For a given ethanol dose, a longer VOM with more lateral branches is associated with a larger LVA [13], which may lead to a more severe inflammatory response and a consequently higher risk of PE. Technological advancements and procedural refinements have been implemented to mitigate this risk. One previous study specifically investigated delayed PE following ethanol infusion and found that reduced ethanol dosage and slower infusion rates were associated with decreased complication rates [14]. Mapping the LVA after VOM‐EI helped identify patients at higher risk for acute PE, guiding post‐procedural management such as intensified monitoring.

The high incidence of acute PE (25.3%) in our cohort raises important safety concerns regarding the nearly fixed, high‐dose ethanol strategy (median 10.0 mL) we used. Acute PE is a multi‐factorial event. While higher ethanol doses typically result in larger LVA [15], this anatomical effect is self‐limiting. Once the VOM branches are fully saturated, any additional ethanol cannot further expand the LVA. Instead, we hypothesize that this excess ethanol may leak into the pericardial space or reflux via collaterals, triggering an unquantifiable chemical inflammatory response. This limitation in measuring total tissue irritation explains why LVA only demonstrated a moderate predictive value for PE (AUC 0.681).

To optimize procedural safety, our findings suggest transitioning from a fixed‐volume ethanol protocol to an individualized dosing strategy. The primary goal should be to administer the minimum effective dose necessary for therapeutic endpoints. Given the potential for ethanol spillover, dosing should be guided primarily by the patient's VOM anatomy as seen on venography. Smaller, less branched veins require less ethanol to achieve tissue saturation, and using a standard high dose in these patients unnecessarily increases the risk of PE. Operators should consider titrating the dose in small increments (e.g., 2–4 mL aliquots) based on the specific vessel characteristics [16]. Additionally, lower doses and extra caution are recommended for highly vulnerable patients—such as those with end‐stage renal disease on dialysis, low body weight, advanced age, or baseline heart failure—as they are less likely to tolerate severe inflammatory responses or pericardial fluid accumulation.

Patients with heart failure often exhibit compromised hemodynamic reserve and reduced cardiac compliance. These factors may diminish their tolerance to even small pericardial fluid accumulations and potentially amplify the inflammatory response to ablation [17]. The pathophysiological interplay between AF and heart failure, characterized by elevated left atrial pressures, atrial fibrosis, and ventricular dysfunction, may predispose these patients to procedural complications, including PE [18]. The development of PE in these patients can be associated with significant morbidity and mortality, particularly if it progresses to cardiac tamponade. This underscores the critical importance of meticulous patient monitoring and refined procedural technique.

In our study, although heart failure was significant in univariate analysis, it lost significance after adjustment for LVA. This attenuation likely reflects the fact that baseline heart failure status is clinically modifiable. Patients typically undergo medical optimization prior to ablation, which mitigates the hemodynamic impact of their admission status. Conversely, LVA serves as a direct surrogate for the extent of chemical tissue injury and subsequent inflammation, acting as a more potent driver of PE than background susceptibility. This mechanistic hierarchy was corroborated by the Boruta algorithm, which definitively ranked LVA as an ’Important’ predictor while relegating heart failure to a ’Tentative’ classification.

While VOM‐EI has demonstrated efficacy in improving rhythm control and reducing arrhythmia burden [19], its potential benefits must be carefully weighed against the risk of complications. The clinical implications of our findings are substantial, as LVA mapping may help stratify patients by PE risk, enabling targeted monitoring and early intervention strategies. It is important to note that our study excluded patients with preoperative PE, VOM dissection, VOM perforation, or CS dissection. However, technical aspects of the procedure itself may also influence PE rates, and the associated inflammatory response can increase the risk of progression to pericardial tamponade.

## 6 Limitations

Despite providing valuable insights, this study has several limitations that warrant consideration. Its retrospective design introduces the potential for selection bias and unmeasured confounding factors that could influence the observed associations. The single‐center nature limits the generalizability of the findings. The sample size was limited (22 outcome events). However, the events‐per‐variable ratio (7.3) is considered acceptable for exploratory analysis, and the results were further corroborated by the Boruta algorithm. The lack of an radiofrequency ablation‐only control group limits our ability to isolate the specific contribution of VOM‐EI to pericardial effusion from the baseline risks of a vulnerable substrate. Our findings aim to identify risk predictors within this specific treatment strategy rather than comparing it to other modalities. Due to the retrospective nature of this study, follow‐up was limited to the immediate postoperative period (≤ 12–24 h) as per standard protocol. Consequently, this study focuses specifically on acute‐phase changes and likely underestimates the true incidence of late‐onset pericardial effusion or peak inflammatory markers (e.g., day 2–3 h‐CRP), which were not systematically captured. Furthermore, the study did not account for potential variations in ethanol infusion techniques, which have been shown to affect complication rates [14]. The moderate discriminatory ability of the low‐voltage area (AUC 0.681) suggests that while it is a significant predictor, other factors likely contribute to the risk of PE.

## 7 Conclusions

In conclusion, while VOM‐EI is a valuable adjunctive strategy for AF ablation, the potential for pericardial effusion remains a clinical concern. Our study identifies the extent of the LVA as a significant independent predictor of acute PE. Importantly, LVA serves as a surrogate for the magnitude of transmural tissue injury, reflecting the interaction between ethanol volume and VOM anatomy, and should be interpreted in the context of underlying patient characteristics.

## Funding

The authors have nothing to report.

## Conflicts of Interest

The authors declare no conflicts of interest.

## Data Availability

Research data are not shared.
